# Increased Expression of CCN2, Epithelial Membrane Antigen, and Fibroblast Activation Protein in Hepatocellular Carcinoma with Fibrous Stroma Showing Aggressive Behavior

**DOI:** 10.1371/journal.pone.0105094

**Published:** 2014-08-15

**Authors:** Gi Jeong Kim, Hyungjin Rhee, Jeong Eun Yoo, Jung Eun Ko, Jee San Lee, Hyunki Kim, Jin Sub Choi, Young Nyun Park

**Affiliations:** 1 Department of Pathology, Yonsei University College of Medicine, Seoul, Republic of Korea; 2 BK21 PLUS Project for Medical Science, Yonsei University College of Medicine, Seoul, Republic of Korea; 3 Department of Surgery, Yonsei University College of Medicine, Seoul, Republic of Korea; 4 Severance Biomedical Science Institute, Yonsei University College of Medicine, Seoul, Republic of Korea; 5 Integrated Genomic Research Center for Metabolic Regulation, Yonsei University College of Medicine, Seoul, Republic of Korea; Boston University Goldman School of Dental Medicine, United States of America

## Abstract

Tumor behavior is affected by the tumor microenvironment, composed of cancer-associated fibroblasts (CAFs). Meanwhile, hepatocellular carcinomas (HCC) with fibrous stroma reportedly exhibit aggressive behavior suggestive of tumor-stroma interaction. However, evidence of the crosstalk remains unclear. In this study, CCN2, epithelial membrane antigen (EMA), fibroblast activation protein (FAP), and keratin 19 (K19) expression was studied in 314 HCCs (cohort 1), 42 scirrhous HCCs (cohort 2), and 36 chronic hepatitis/cirrhosis specimens by immunohistochemistry. Clinicopathological parameters were analyzed according to the expressions of these markers. In tumor epithelial cells from cohort 1, CCN2 and EMA were expressed in 15.3% and 17.2%, respectively, and their expressions were more frequent in HCCs with fibrous stroma (≥5% of tumor area) than those without (P<0.05 for all); CCN2 expression was well correlated with K19 and EMA expression. In tumor stromal cells, FAP expression was found in 6.7%. In cohort 2, CCN2, EMA, and FAP expression was noted in 40.5%, 40.5%, and 66.7%, respectively, which was more frequent than that in cohort 1 (P<0.05 for all). Additionally, EMA expression was associated with the expression of K19, CCN2, and FAP (P<0.05 for all); EMA expressing tumor epithelial cells showed a topographic closeness to FAP-expressing CAFs. Analysis of disease-free survival revealed CCN2 expression to be a worse prognostic factor in both cohort 1 (*P* = 0.005) and cohort 2 (*P* = 0.023), as well as EMA as a worse prognostic factor in cohort 2 (*P* = 0.048). In conclusion, expression of CCN2, EMA, and FAP may be involved in the activation of CAFs in HCC, giving rise to aggressive behavior. Significant correlation between EMA-expressing tumor cells and FAP-expressing CAFs and their topographic closeness suggests possible cross-talk between tumor epithelial cells and stromal cells in the tumor microenvironment of HCC.

## Introduction

The biological behavior of tumors is reportedly affected by not only malignant tumor cells themselves but also by the tumor microenvironment including tumor stroma [1]–[3]. The tumor stroma is a complicated system that consists of signaling molecules, extracellular matrix proteins, proteolytic enzymes, blood vessels, and a variety of cellular components, such as cancer-associated fibroblasts (CAFs) and immune cells [4], [5]. CAFs in tumor stroma are histologically categorized as myofibroblasts or activated fibroblasts, and they have been reported to be associated with aggressive biological behavior, poor prognosis, and resistance to chemotherapy and radiation therapy in breast cancer, pancreatic cancer, and colon cancer [6]–[10]. Therefore, CAFs could influence the biological characteristics of tumor cells through tumor-stroma cross-talk. However, crosstalk between tumor cells and activated fibroblasts has not been fully explored in HCCs.

Hepatocellular carcinoma (HCC) is the seventh most common malignancy worldwide and the third greatest cause of cancer related mortality, especially in Asia and sub-Saharan Africa [11]. Most HCCs contain no or only little amounts of fibrous stroma; nevertheless, some HCCs without history of preoperative treatment exhibit various amounts of fibrous stroma between tumor nests. In a previous study, we showed that HCC specimens with abundant fibrous stroma, known as scirrhous HCC, exhibit an aggressive biological behavior and the expression of “stemness”-related markers, along with activation of TGF-*β* signature and epithelial-mesenchymal transition (EMT)-related genes [12].

CCN2 (previously known as connective tissue growth factor, IGFBP8, etc.), a fibrogenic cytokine, is involved in virtually all fibrotic pathologies, both benign and malignant [13]. Recently, CCN2 expression was reported to be impeded by TGF-*β* receptor inhibition, resulting in a decrease of the stromal components in HCC [14]. Epithelial membrane antigen (EMA) is a member of a family of transmembrane mucin glycoproteins, with a high carbohydrate content and extensive O-linked glycosylation of its extracellular domain [15]. Recently, EMA mRNA was reported to be up-regulated in a co-culture study of hepatoma cells and activated hepatic stellate cells (HSCs), compared to stromal cells cultured alone [16]. Furthermore, clinical studies have reported a relationship between EMA expression and poor prognosis in various malignant tumors, including lung cancer, gastric cancer, gallbladder cancer, and HCC [17]–[20]. Fibroblast activation protein (FAP), a member of the serine protease family, has been reported to increase stromal cell proliferation and invasiveness, as well as reduce cell apoptosis [21]. FAP is also recognized as a useful marker of CAFs, selectively expressed in fibroblasts of several epithelial cancers, and is reported to be related to worse prognosis of pancreatic adenocarcinoma and colon cancer [21]–[24].

Concerning the tumor microenvironment of HCCs, the molecular mechanism involved in the formation of tumor fibrous stroma and tumor-stroma cross-talk remains unclear. Thus, we attempted to evaluate the expressions of CCN2, EMA, and FAP and their correlation with clinicopathological features of HCCs. As well, their topographic expression patterns were further examined in HCCs with abundant fibrous stroma (scirrhous HCCs).

## Materials and Methods

### Patients and clinicopathological analysis

The HCC specimens included in this study were morphologically typical of HCC, and cases that could be classified as combined hepatocellular-cholangiocarcinoma or with a history of preoperative treatment were excluded. Formalin-fixed, paraffin-embedded HCC specimens were obtained from the archives of the Department of Pathology, Severance Hospital, Yonsei University College of Medicine. Liver specimens were provided by the Liver Cancer Specimen Bank, National Research Resource Bank program of the Korea Science and Engineering Foundation of the Ministry of Science and Technology. This study was approved by the Institutional Review Board of Severance Hospital, Yonsei University College of Medicine (Seoul, Korea). The Institutional Review Board waived the need for consent (4-2013-0912).

This study was performed in specimens from two cohorts of patients with HCC. Cohort 1 consisted of 314 cases of HCC from January 2007 to March 2011; there were 254 males and 60 females, ranging in age from 28 to 81 years (55.6±10.1, mean ±SD). To investigate the spatial relationship between epithelial cells and CAFs, cohort 2 included 42 cases of HCC with abundant fibrous stroma (≥50% of the tumor area) (scirrhous HCC). The specimens were obtained from September 2001 to December 2010, there were 29 males and 13 females, whose ages ranged from 27 to 71 years (range, 53.7±8.3, mean ±SD). Twenty-five cases were included in both of cohort 1 and 2. As a control group, 36 non-tumor tissues of chronic hepatitis/cirrhosis were studied.

Histopathologic analysis was performed for both cohorts on whole sections of representative tissue blocks. For each case, tumor size, differentiation according to Edmondson-Steiner grade, tumor capsule formation, lymphovascular invasion, multiplicity of tumors, presence of fibrous stroma, and pathology of non-neoplastic liver were recorded. In cohort 1, presence of fibrous stroma was defined as fibrotic areas ≥5% of the tumor area.

Clinical data from each patient were obtained from a careful review of their medical records, including hepatitis B virus surface antigen status, hepatitis C virus antibody, and tumor-node-metastasis (TNM) classification according to the 7th American Joint Committee on Cancer/International Union against Cancer (AJCC/UICC) staging system.

### Tissue microarray construction

A representative formalin-fixed, paraffin-embedded block containing HCC was selected for each of the 314 available cases of cohort 1. The arrays were constructed in triplicate using a 3-mm punch on a tissue-arraying instrument (Beecher Instruments, Silver Springs, FL, USA). The cases were reviewed on conventional hematoxylin and eosin-slides, and representative areas were marked on each slide. Using a marker pen, the corresponding region was circled on the “donor” paraffin block. The samples were then arrayed on to a “recipient” block.

### Immunohistochemistry

Immunohistochemical stain was performed using tissue microarrays from cohort 1 and paraffin-embedded whole tissue sections for topographic assessment from cohort 2. To compare the phenotypical characteristics between CAFs of tumor fibrous stroma and fibroblasts in benign fibrous stroma, 36 cases of chronic hepatitis or cirrhosis, which included at least 10 portal tracts, were also immunostained.

The primary antibodies used were anti- CCN2 (1∶300, Abcam, Cambridge, UK), anti-EMA (1∶100, Dako, Glostrup, Denmark), anti-FAP (D8, 1∶100, Vitatex, Stony Brook, NY, USA), and Keratin 19 (K19) (1∶100, Dako). Immunohistochemical stain for CCN2, EMA, and K19 was performed using automated staining system (Discovery XT, Ventana Medical Systems, AZ, USA), and that for FAP was performed using horseradish peroxidase-conjugated ant-rat immunoglobulin and Envision kit (DAKO) according to the manufacturer's instructions. All immunohistochemical markers were assessed by a light microscope. The immunohistochemical staining results were interpreted in a semiquantitative way and given a staining score, from 0 to 3, as follows: 0, staining in <5% of tumor cells; 1, weak staining in ≥5%; 2, moderate staining in ≥5%; and 3, strong staining in ≥5% of the tumor cells. Positive staining was defined as a staining score of 2 or 3, whereas scores of 0 and 1 were regarded as negative. Two pathologists (G. J. Kim and Y. N. Park) assessed the staining results without awareness of the clinicopathological data for each case.

### Statistical analysis

Statistical analyses were performed using SPSS software version 20.0 for Windows (SPSS Inc., Chicago, IL, USA). Categorical variables were analyzed by the chi-square test or Fisher's exact test. On survival analysis, clinicopathologic variables were dichotomized and analyzed according to their effect on prognosis. Disease-free survival (DFS) and overall survival (OS) analysis was performed using the Kaplan–Meier method, and differences between the groups were assessed using the log-rank test. Univariate and multivariate survival analyses were carried out using Cox proportional hazard regression models. Only variables significant in the univariate analysis of factors affecting survival were used in the stepwise multivariate analysis. Estimated relative risks of death were expressed as adjusted hazard ratios (HR) and corresponding 95% confidence intervals (CI). Statistical significance was assumed for *P*-values <0.05.

## Results

### CCN2, EMA, and FAP expression and the clinicopathological characteristics of HCC (Cohort 1, n = 314)

**Figure 1:**
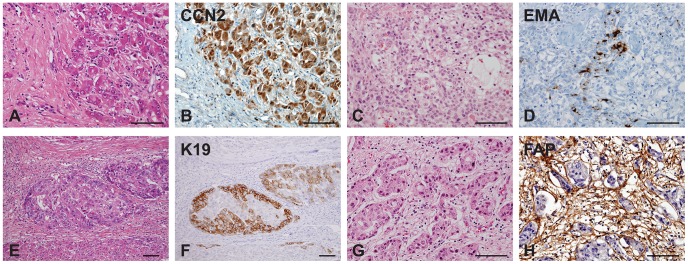
Connective tissue growth factor (CCN2), epithelial membrane antigen (EMA), fibroblast activation protein (FAP), and keratin 19 (K19) expression in hepatocellular carcinomas (HCCs) of cohort 1. A) Small nests of tumor cells are surrounded by fibrous stroma. B) Immunohistochemistry for CCN2 exhibits diffuse cytoplasmic expression in tumor epithelial cells of HCC. C–D) Immunohistochemistry for EMA reveals patchy or focal expression pattern in tumor epithelial cells of HCC. E–F) K19 is focally positive in the periphery of tumor nests. (G–H) FAP is expressed in the cytoplasm of cancer-associated fibroblasts of tumor stroma. (Scale bars represent 100 µm.)

To assess the expressions of CCN2, EMA, K19, and FAP in HCCs in regards to their clinicopathologic significance, we investigated these markers by immunohistochemistry of tissue microarrays from 314 cases of HCC (cohort 1). Positive expression rates of CCN2, EMA, K19, and FAP were 15.3% (48/314), 17.2% (54/314), 22.3% (70/314), and 6.7% (21/314), respectively. CCN2, EMA, and K19 expression was noted in tumor epithelial cells, but not in CAFs. CCN2 was diffusely expressed throughout the tumor cells upon cytoplasmic staining (Figure 1. A–B). Expression of EMA, on the contrary, was either patchy or diffuse in the tumor cells upon membranous and/or cytoplasmic staining (Figure 1. C–D). K19 was focally expressed in the tumor cells upon cytoplasmic and/or membranous staining (Figure 1. E–F). FAP was, on the other hand, expressed in the cytoplasm of CAFs, but not in tumor epithelial cells (Figure 1. G–H).

The expressions of CCN2, EMA, and FAP were evaluated according to clinicopathologic parameters of HCC (Table 1). CCN2 expression was significantly related to background cirrhosis (*P* = 0.035), absence of tumor capsule (*P* = 0.049), and presence of tumor fibrous stroma in HCC (*P* = 0.028). EMA expression demonstrated a significant association with presence of tumor fibrous stroma only (*P* = 0.003). Expression of CCN2 was significantly correlated with K19 immunoreactivity (*P* = 0.018), whereas EMA expression was not. FAP expression was significantly correlated with a solitary tumor mass (*P* = 0.031), but it was not significantly correlated with any other clinicopathologic parameter (*P*>0.05).

**Table 1:** Clinicopathological characteristics of HCCs according to CCN2, EMA, and FAP expression in cohort 1 (*n* = 314).

	CCN2 (in tumor epithelial cells)			EMA (in tumor epithelial cells)			FAP (in tumor stromal cells)		
	Positive (%)	Negative (%)	*P*	Positive (%)	Negative (%)	*P*	Positive (%)	Negative (%)	*P*
**Frequency**	48 (15.3)	266 (84.7)		54 (17.2)	260 (82.8)		21 (6.7)	293 (93.3)	
**Sex**			0.259			0.795			0.254
Female	12 (25.0)	48 (18.0)		11 (20.4)	49 (18.8)		6 (28.6)	54 (18.4)	
Male	36 (75.0)	218 (82.0)		43 (79.6)	211 (81.2)		15 (71.4)	239 (81.6)	
**Age (years)**			0.328			0.494			0.207
<55	18 (37.5)	120 (45.1)		26 (48.1)	112 (43.1)		12 (57.1)	126 (43.0)	
≥55	30 (62.5)	146 (54.9)		28 (51.9)	148 (56.9)		9 (42.9)	167 (57.0)	
**Etiology**			0.697			0.112			0.089
Non-viral	5 (10.4)	33 (12.4)		10 (18.5)	28 (10.8)		5 (23.8)	33 (11.3)	
HBV	39 (81.3)	220 (82.7)		41 (75.9)	218 (83.8)		16 (76.2)	243 (82.9)	
HCV	4 (8.3)	13 (4.9)		3 (5.6)	14 (5.4)		0 (0.0)	17 (5.8)	
**Cirrhosis**			0.035			0.169			0.495
Absent	15 (31.2)	127 (47.7)		29 (53.7)	113 (43.5)		11 (52.4)	131 (44.7)	
Present	33 (68.8)	139 (52.3)		25 (46.3)	147 (56.5)		10 (47.6)	162 (55.3)	
**Tumor size (cm)**			0.388			0.138			0.586
<5	36 (75.0)	214 (80.5)		39 (72.2)	211 (81.2)		18 (85.7)	232 (79.2)	
≥5	12 (25.0)	52 (19.5)		15 (27.8)	49 (18.8)		3 (14.3)	61 (20.8)	
**Edmondson grade**			0.141			0.844			0.128
I/II	19 (39.6)	136 (51.1)		26 (48.1)	129 (49.6)		7 (33.3)	148 (50.5)	
III/IV	29 (60.4)	130 (48.9)		28 (51.9)	131 (50.4)		14 (66.7)	145 (49.5)	
**Capsule formation**			0.049			0.141			0.406
Absent	13 (27.1)	41 (15.4)		13 (24.1)	41 (15.8)		5 (23.8)	49 (16.7)	
Present	35 (72.9)	225 (84.6)		41 (75.9)	219 (84.2)		16 (76.2)	244 (83.3)	
**Vascular invasion**			0.604			0.400			0.990
Absent	19 (39.6)	116 (43.6)		26 (48.1)	109 (41.9)		9 (42.9)	126 (43.0)	
Present	29 (60.4)	150 (56.4)		28 (51.9)	151 (58.1)		12 (57.1)	167 (57.0)	
**Multiplicity**			0.198			0.705			0.031
Single	37 (77.1)	225 (84.6)		46 (85.2)	216 (83.1)		21 (100.0)	241 (82.3)	
Multiple	11 (22.9)	41 (15.4)		8 (14.8)	44 (16.9)		0 (0.0)	52 (17.7)	
**Stage (by AJCC)**			0.096			0.747			0.615
I–II	43 (89.6)	254 (95.5)		52 (96.3)	245 (94.2)		21 (100.0)	276 (94.2)	
III–IV	5 (10.4)	12 (4.5)		2 (3.7)	15 (5.8)		0 (0.0)	17 (5.8)	
**Fibrous stroma**			0.028			0.003			0.219
Absent	36 (75.0)	232 (87.2)		39 (72.2)	229 (88.1)		16 (76.2)	252 (86.0)	
Present	12 (25.0)	34 (12.8)		15 (27.8)	31 (11.9)		5 (23.8)	41 (14.0)	
**K19 expression**			0.018			0.730			0.863
Negative	31 (64.6)	213 (80.1)		41 (75.9)	203 (78.1)		16 (76.2)	228 (77.8)	
Positive	17 (35.4)	53 (19.9)		13 (24.1)	57 (21.9)		5 (23.8)	65 (22.2)	

HCC, Hepatocellular carcinoma; CCN2, Connective tissue growth factor; EMA, Epithelial membrane antigen; FAP, Fibroblast activation protein.

Expression rates of CCN2 and EMA were significantly correlated with each other (*P* = 0.001). Among 48 cases that exhibited CCN2 expression, 16 cases (33.3%, 16/48) were positive for EMA. In the CCN2 negative cases, however, the expression rate of EMA was relatively low (14.3%, 38/266). The expressions of CCN2 and FAP showed no significant correlation with each other (*P* = 0.752); two cases (4.2%, 2/48) were positive for FAP in the CCN2 positive group and 19 cases (7.1%, 19/266) were positive for FAP in the CCN2 negative group. As well, the expressions of EMA and FAP also showed no significant correlation with each other (*P* = 0.406); five cases (9.3%, 5/54) were positive for FAP in the EMA positive group and 16 cases (6.2%, 16/260) were positive for FAP in the EMA negative group.

### CCN2, EMA, and FAP expression and the clinicopathological characteristics of HCCs with abundant fibrous stroma (scirrhous HCC) (Cohort 2, n = 42)

The expressions of CCN2 and EMA were significantly correlated with each other and also with the presence of tumor fibrous stroma in cohort 1. These results might imply cross-talk between CCN2 and EMA expression and tumor fibrous stromal components. As most HCCs of cohort 1 showed no or little tumor fibrous stroma, we evaluated the expressions of these markers in another cohort of scirrhous HCC, which had abundant (≥50% of tumor area) fibrous stroma. Whole tumor sections from representative paraffin-embedded tissue blocks were used to assess the topographic expression patterns of CCN2, EMA, K19, and FAP in scirrhous HCCs. Therein, the positive expression rates of CCN2, EMA, K19, and FAP were 40.5% (17/42), 40.5% (17/42), 33.3% (14/42), and 66.7% (28/42), respectively. The expression rates of CCN2, EMA, K19, and FAP in cohort 2 were significantly higher than those in cohort 1 (P<0.001, P = 0.001, P = 0.013, P<0.001, respectively).

**Figure 2:**
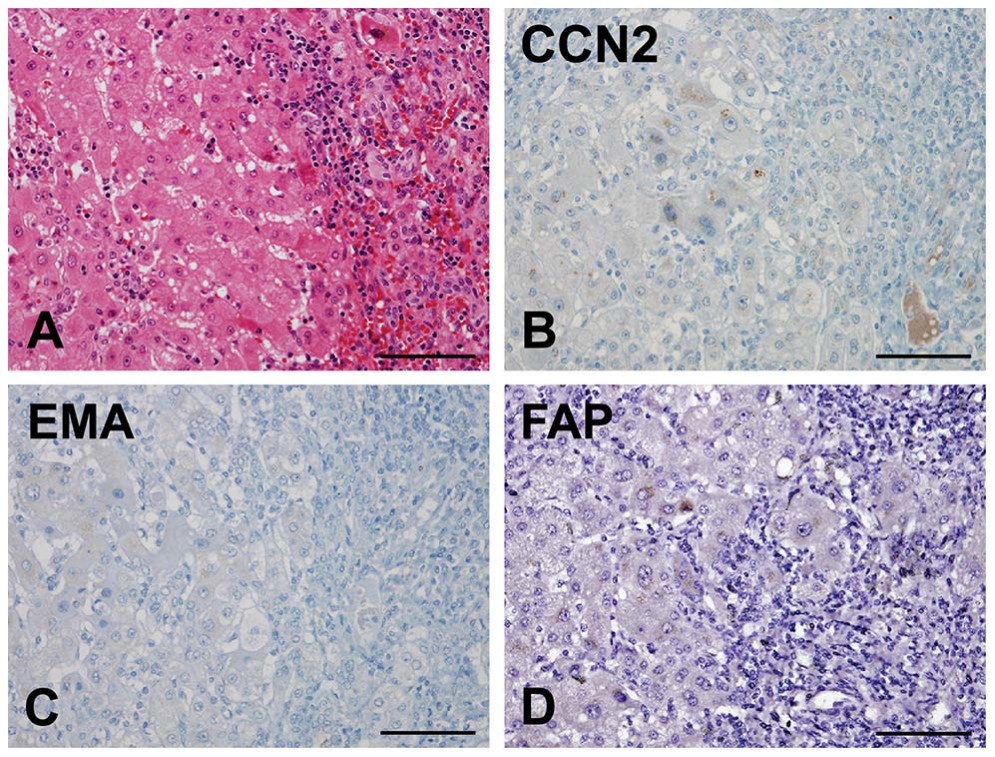
Connective tissue growth factor (CCN2), epithelial membrane antigen (EMA), fibroblast activation protein (FAP), and keratin 19 (K19) expression in liver cirrhosis. A) Liver cirrhosis showing regenerative hepatic nodules and fibrotic septa with chronic inflammatory cells. CCN2 (B), EMA (C), and FAP (D) expression is negative in both hepatocytes and benign stromal cells from cirrhosis specimens. (Scale bars represent 100 µm.)

In comparison of tumor fibrous stroma of HCC and benign fibrous stroma of the liver, the expressions of CCN2, EMA, and FAP were investigated in specimens of chronic hepatitis/cirrhosis (n = 36) (Figure 2). CCN2 and EMA were not detected in either stromal cells or hepatocytes of chronic hepatitis/cirrhosis specimens. Additionally, FAP was not expressed in the stromal cells of most cases of chronic hepatitis/cirrhosis, and it was only focally detected in stromal cells from three cases (3/36, 8.3%) of chronic hepatitis/cirrhosis. Moreover, FAP was not noted in any benign hepatocytes for all cases. Accordingly, FAP expression was significantly higher in CAFs from HCCs than in benign fibrous stroma from chronic hepatitis/cirrhosis specimens (*P* = 0.012).

The EMA expression rate was significantly higher in HCCs with CCN2 expression, compared to those without (*P* = 0.046). Ten cases (58.8%, 10/17) were positive for EMA among CCN2 positive specimens, while EMA was expressed in only seven (28.0%, 7/25) CCN2 negative specimens. Moreover, the EMA expression rate was also significantly higher in HCCs with FAP expression, compared to those without (*P* = 0.020), in this cohort. Fifteen cases (53.6%, 15/28) were positive for EMA among FAP positive specimens, while EMA was expressed in only two (14.3%, 2/14) FAP negative specimens. Additionally, EMA expression was associated with K19 expression; K19 was positive in 52.9% (9/17) of EMA positive cases and negative in 20.0% (5/25) of EMA negative cases (*P* = 0.026). The expressions of CCN2 and FAP showed no significant correlation with each other (*P* = 0.331); 13 cases (76.5%, 13/17) were positive for FAP among CCN2 positive specimens and 15 cases (60.0%, 15/25) were positive for FAP among CCN2 negative specimens.

**Figure 3:**
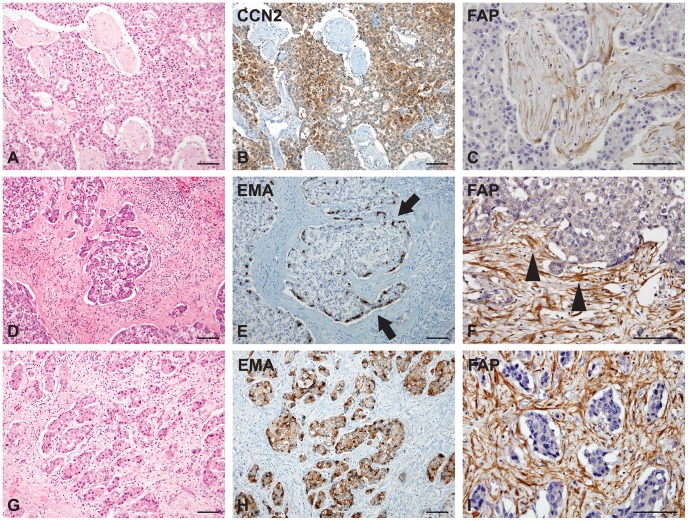
Connective tissue growth factor (CCN2), epithelial membrane antigen (EMA), and fibroblast activation protein (FAP) expression in scirrhous hepatocellular carcinomas (HCCs with abundant fibrous stroma) of cohort 2. A–C) CCN2 (B) is diffusely expressed in the nests of tumor epithelial cells, and the tumor stromal cells between the nests of tumor epithelial cells exhibit strong FAP expression (C). D–F) EMA is mainly expressed in the periphery (E, arrows) of large tumor nests in contact with FAP-expressing cancer-associated fibroblasts (CAFs) of tumor fibrous stroma (F, arrowheads). G–I) HCCs with small nests or a trabecular pattern show diffuse expression for EMA in the tumor epithelial cells (H), which are closely admixed with FAP-expressing CAFs of tumor stroma. (Scale bars represent 100 µm.)

CCN2 was diffusely expressed in tumor epithelial cells without a specific pattern and the CCN2-expressing tumor cells were closely surrounded by tumor stroma with FAP-expressing CAFs (Figure 3. A–C). EMA exhibited focal or diffuse expression in the cytoplasm and/or membranes of tumor epithelial cells. The topographical expression pattern thereof was further assessed in 17 cases positive for EMA expression, including six cases of a large tumor nest pattern and 11 cases of a small tumor nest/trabecular pattern. Interestingly, EMA expression patterns demonstrated topographic closeness between the tumor epithelial cells and CAFs of tumor stroma: EMA expression was noted at the periphery of tumor nests, where the tumor cells were more closely in contact with the FAP-expressing tumor stroma in 50% (3/6) of cases with a large tumor nest pattern (Figure 3. D–F). Meanwhile, all 11 cases of a small nest/trabecular pattern showed diffuse expression of EMA, and tumor cells expressing EMA were surrounded by FAP-expressing tumor stroma (Figure 3. G–I).

The clinicopathological features of HCC according to the expressions of CCN2, EMA, and FAP in HCCs with fibrous stroma are summarized in Table 2. CCN2 expression rate was significantly higher in large tumors (≥5 cm), compared to small ones (<5 cm) (*P* = 0.023). FAP expression rate was significantly higher in HCCs with vascular invasion, compared to those without (*P* = 0.030).

**Table 2:** Clinicopathological characteristics of scirrhous HCCs according to CCN2, EMA, and FAP expression in cohort 2 (*n* = 42).

	CCN2 (in tumor epithelial cells)			EMA (in tumor epithelial cells)			FAP (in tumor stromal cells)		
	Positive (%)	Negative (%)	*P*	Positive (%)	Negative (%)	*P*	Positive (%)	Negative (%)	*P*
**Frequency**	17 (40.5)	25 (59.5)		17 (40.5)	25 (59.5)		28 (66.7)	14 (33.3)	
**Sex**			0.859			0.505			0.238
Female	5 (29.4)	8 (32.0)		4 (23.5)	9 (36.0)		7 (25.0)	6 (42.9)	
Male	12 (70.6)	17 (68.0)		13 (76.5)	16 (64.0)		21 (75.0)	8 (57.1)	
**Age (years)**			0.753			0.346			1.000
<55	8 (47.1)	13 (52.0)		10 (58.8)	11 (44.0)		14 (50.0)	7 (50.0)	
≥55	9 (52.9)	12 (48.0)		7 (41.2)	14 (56.0)		14 (50.0)	7 (50.0)	
**Etiology**			1.000			1.000			1.000
Non-viral	3 (17.6)	5 (20.0)		3 (17.6)	5 (20.0)		5 (17.9)	3 (21.4)	
HBV	13 (76.5)	20 (80.0)		14 (82.4)	19 (76.0)		22 (78.6)	11 (78.6)	
HCV	1 (5.9)	0 (0.0)		0 (0.0)	1 (4.0)		1 (3.5)	0 (0.0)	
**Cirrhosis**			0.542			0.963			1.000
Absent	10 (58.8)	17 (68.0)		11 (64.7)	16 (64.0)		18 (64.3)	9 (64.3)	
Present	7 (41.2)	8 (32.0)		6 (35.3)	9 (36.0)		10 (35.7)	5 (35.7)	
**Tumor size (cm)**			0.023			0.324			0.653
<5	7 (41.2)	19 (76.0)		9 (52.9)	17 (68.0)		18 (64.3)	8 (57.1)	
≥5	10 (58.8)	6 (24.0)		8 (47.1)	8 (32.0)		10 (35.7)	6 (42.9)	
**Edmondson grade**			0.346			0.116			0.100
I/II	7 (41.2)	14 (56.0)		6 (35.3)	15 (60.0)		11 (39.3)	10 (71.4)	
III/IV	10 (58.8)	11 (44.0)		11 (64.7)	10 (40.0)		17 (60.7)	4 (28.6)	
**Capsule formation**			0.158			1.000			0.545
Absent	2 (11.8)	0 (0.0)		1 (5.9)	1 (4.0)		2 (7.1)	0 (0.0)	
Present	15 (88.2)	25 (100.0)		16 (94.1)	24 (96.0)		26 (92.9)	14 (100.0)	
**Vascular invasion**			0.731			0.731			0.030
Absent	4 (23.5)	8 (32.0)		4 (23.5)	8 (32.0)		5 (17.9)	7 (50.0)	
Present	13 (76.5)	17 (68.0)		13 (76.5)	17 (68.0)		23 (82.1)	7 (50.0)	
**Multiplicity**			0.286			1.000			0.590
Single	14 (82.4)	24 (96.0)		15 (88.2)	23 (92.0)		26 (92.9)	12 (85.7)	
Multiple	3 (17.6)	1 (4.0)		2 (11.8)	2 (8.0)		2 (7.1)	2 (14.3)	
**Stage (by AJCC)**			0.556			1.000			0.254
I–II	15 (88.2)	24 (96.0)		16 (94.1)	23 (92.0)		27 (96.4)	12 (85.7)	
III–IV	2 (11.8)	1 (4.0)		1 (5.9)	2 (8.0)		1 (3.6)	2 (14.3)	
**K19 expression**			0.824			0.026			0.313
Negative	11 (64.7)	17 (68.0)		8 (47.1)	20 (80.0)		17 (60.7)	11 (78.6)	
Positive	6 (35.3)	8 (32.0)		9 (52.9)	5 (20.0)		11 (39.3)	3 (21.4)	

HCC, Hepatocellular carcinoma; CCN2, Connective tissue growth factor; EMA, Epithelial membrane antigen; FAP, Fibroblast activation protein.

### Prognostic significance of CCN2, EMA, and FAP expression

All HCC patients underwent curative resection, and the mean follow-up times were 31.2 months (range, 0–76) in cohort 1 and 44.6 months (range, 8–138) in cohort 2.

**Figure 4:**
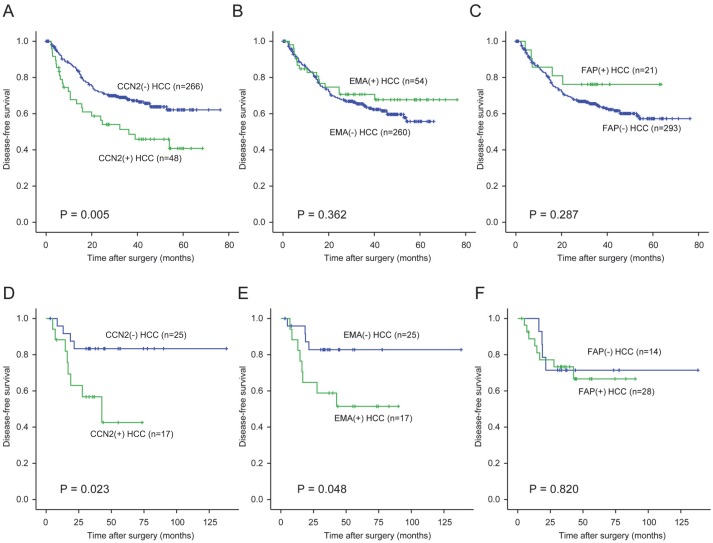
Disease-free survival (DFS) analysis of hepatocellular carcinoma (HCC) patients according to the expressions of connective tissue growth factor (CCN2), epithelial membrane antigen (EMA), and fibroblast activation protein (FAP). A–C) DFS analysis of HCC patients in cohort 1. HCC patients with positive expression of CCN2 (A) exhibit a significantly worse DFS curve compared to those without (*P* = 0.005). There is no significant difference in DFS rate according expression of EMA (B) or FAP (C). D–F) DFS analysis of scirrhous HCC patients (cohort 2). CCN2 (D) and EMA (E) expression significantly influences DFS rates among scirrhous HCC patients (*P* = 0.023 and *P* = 0.048, respectively), whereas there is no significant difference in DFS rate according to FAP expression (F).

Among the 314 HCC patients of cohort 1, DFS rate was significantly lower in HCC patients expressing CCN2, compared to those that did not (*P* = 0.005, Figure 4A); EMA and FAP expression was not associated with patient outcomes in this cohort (*P* = 0.362 and *P* = 0.287, respectively) (Figure 4. B–C). Univariable analysis revealed background cirrhosis (*P* = 0.002), large tumor size (≥5 cm) (*P* = 0.001), high Edmondson grade (*P* = 0.007), vascular invasion (*P*<0.001), multiple tumors (*P*<0.001), tumor stage III/IV (*P*<0.001), and CCN2 expression (*P* = 0.005) to be adverse prognostic factors for DFS after surgery. Subsequent multivariable analysis indicated background cirrhosis (HR = 1.815, *P* = 0.004) and vascular invasion (HR = 1.764, *P* = 0.015) as independent prognostic factors for DFS after surgery; CCN2 expression was not significant in multivariate analysis (HR = 1.561, *P* = 0.056, Table 3). Additionally, OS rate was not significant different according to expression of these markers (*P*>0.05).

**Table 3:** Univariate and multivariate analysis of disease-free survival rate for HCC in cohort 1.

	Univariate			Multivariate		
	HR	95% CI	*P*	HR	95% CI	*P*
**Sex**						
Female	1					
Male	0.989	0.620–1.579	0.965			
**Age (years)**						
<55	1					
≥55	1.200	0.820–1.755	0.349			
**Etiology**						
Non-viral	1					
Viral	0.939	0.536–1.645	0.825			
**Cirrhosis**						
Absent	1			1		
Present	1.859	1.251–2.761	0.002	1.815	1.208–2.729	0.004
**Tumor size (cm)**						
<5	1			1		
≥5	1.977	1.309–2.986	0.001	1.533	0.952–2.468	0.079
**Edmondson grade**						
I/II	1			1		
III/IV	1.685	1.150–2.469	0.007	1.186	0.779–1.804	0.427
**Vascular invasion**						
Absent	1			1		
Present	2.179	1.437–3.302	<0.001	1.764	1.118–2.784	0.015
**Multiplicity**						
Single	1			1		
Multiple	2.250	1.470–3.445	<0.001	1.399	0.787–2.488	0.253
**Stage (by AJCC)**						
I–II	1			1		
III–IV	4.652	2.636–8.211	<0.001	2.214	0.921–4.898	0.077
**Fibrous stroma**						
Not abundant	1					
Abundant	0.931	0.548–1.581	0.790			
**K19 expression**						
Negative	1					
Positive	0.952	0.609–1.487	0.828			
**CCN2 expression**						
Negative	1			1		
Positive	1.886	1.207–2.948	0.005	1.561	0.989–2.465	0.056
**EMA expression**						
Negative	1					
Positive	0.782	0.460–1.329	0.363			
**FAP expression**						
Negative	1					
Positive	0.617	0.252–1.515	0.292			

HCC, Hepatocellular carcinoma; CCN2, Connective tissue growth factor; EMA, Epithelial membrane antigen; FAP, Fibroblast activation protein.

In cohort 2, consisting of 42 scirrhous HCCs, DFS rates were significantly lower for both CCN2-positive and EMA-positive specimens, compared to negative specimens (*P* = 0.023 and *P* = 0.048, respectively, Figure 4. D–E). Nevertheless, there were no differences in OS rates according to CCN2 and EMA expression (*P* = 0.484 and 0.230, respectively). As well, expression of FAP showed no correlation with DFS (*P* = 0.283, Figure 4F) and OS (*P* = 0.820), respectively.

## Discussion

Tumor behavior is affected by not only malignant tumor cells themselves but also by the tumor microenvironment, including CAFs [2], [4], [5]. Although, HCCs usually show no or only little amounts of fibrous stroma, in our previous study, we found that so called scirrhous HCCs, HCCs with abundant fibrous stroma, exhibit an aggressive biological behavior, along with expression of stemness-related markers and activation of TGF-*β* signature and EMT-related genes [12]. These findings suggest tumor-stroma interaction in HCC; however, the activation mechanisms thereof remain unclear.

FAP was initially identified as being expressed in reactive fibroblasts for embryonic development or in chronic inflammation [25], [26]. More importantly, FAP is recognized as a marker of CAFs, and is reported to increase stromal cell proliferation and invasiveness, reduce cell apoptosis, and to be associated with worse prognosis in colon cancer and pancreas cancer [21]–[24]. The present study found that FAP is expressed predominantly in CAFs from the tumor fibrous stroma of HCCs, and is significantly correlated with frequent vascular invasion in scirrhous HCCs. In contrast, FAP expression was rarely found in benign fibrotic tissue of chronic hepatitis/cirrhosis. These findings suggest that FAP is involved in the activation of CAFs in tumor stroma, which differ from benign fibroblasts in the fibrous tissue of chronic hepatitis/cirrhosis.

An *in vitro* co-culture model study of human hepatoma cells and activated HSCs demonstrated increases in EMA mRNA when those cells were cultured together, compared to culture of stromal cells alone [16]. Our study revealed significantly higher rates of EMA expression in HCCs with fibrous stroma (≥5% of tumor area), compared to those without (cohort 1), and this was related to poor DFS in scirrhous HCC patients (cohort 2). These findings were consistent with previous reports that EMA was a poor prognostic factor in HCC [17], [27]. Interestingly, in HCCs with large tumor nests, EMA expression was higher at the peripheral portions of the tumor nests where tumor cells were more closely in contact with FAP-expressing CAFs. Meanwhile, in HCCs with small nest/trabecular pattern, EMA expression was rather diffuse: the tumor cells closely intermingled with CAFs expressing FAP. This topographic expression pattern that suggests topographic closeness between the EMA-expressing tumor cells and CAFs of tumor stroma, which was similar to that of K19 expression in HCCs with fibrous stroma reported in our previous study [12]. Furthermore, the frequency of EMA expression was shown to be significantly correlated with that of FAP expression in scirrhous HCCs (cohort 2). Taken together, we discerned that EMA and FAP may be important in tumor-stroma cross-talk via activation of CAFs. To our knowledge, this is the first study to verify topographically the expression patterns of EMA in human HCC tissues with activated CAFs.

CCN2 is a fibrogenic cytokine that mediates almost all fibrotic processes [13]. Overexpression of CCN2 in fibroblasts produces large amounts of extracellular matrix and enhances benign fibrotic changes in the pancreas, kidney, lung, and liver [28]–[31]. In addition to benign fibrotic processes, CCN2 overexpression is also known to be responsible for pathologic fibrosis, including desmoplastic reaction in cancer [32]. Inhibition of TGF-*β*, which is typically activated in HCCs with fibrous stroma [12], was reported to downregulate CCN2 and block tumor-stroma cross-talk and tumor progression in HCC [14]. Two prior studies assessing the prognostic effects of CCN2 expression in HCCs disclosed that the expression levels of intra tumoral CCN2 were significantly higher in HCCs with bone metastasis [33], [34]. Moreover, the CCN2 mRNA was expressed in tumor cells of EMT-phenotype in HCC, facilitating migration, invasion, and progression of the tumor cells *in vitro* [35]. In accordance with these studies, we discovered that CCN2 expression is related to more infiltrative growth without tumor capsule and worse DFS in HCCs. Although CCN2 is well known as fibrogenic cytokine, to our knowledge, no study has reported on a relationship between CCN2 expression and tumor fibrous stromal components in HCC. Herein, we demonstrated significant CCN2 expression in HCCs with fibrous stroma (≥5% of the tumor area) (cohort 1) and even greater expression in scirrhous HCCs (fibrous stroma ≥50% of the tumor area) (cohort 2). The expression of CCN2 was correlated with absence of capsule formation, which is a characteristic pathological feature of invasive tumor growth, as well as frequent K19 expression (cohort 1), larger tumor size (cohort 2), and shorter disease free survival (cohort 1 and 2). Furthermore, expression of CCN2 was shown to be associated with EMA expression in both cohorts, which seems to be important in epithelial-stromal interactions in HCC. Taken together, we suggest that CCN2 expression is involved in the activation of CAFs and tumor fibrous stroma formation, which is related to the aggressive biological behavior of HCC. [12]

Interestingly, CCN2 expression was well correlated with K19 expression in the HCC specimens of this study. We previously reported that HCCs expressing stemness-related markers, such as K19, exhibited greater formation of fibrous stroma, more vascular invasion, and more aggressive clinical outcomes upon activation of EMT-related genes [36]. The correlations between CCN2, K19, and fibrous stroma are of interest, in that they might imply that stemness is regulated by tumor stroma, as in various other tumors. [37]–[39] Accordingly, the underlying molecular mechanisms thereof should be further investigated, as delineating the microenvironmental regulation of stemness might provide new targets for cancer therapy. [40]

In conclusion, the expressions of CCN2, EMA, and FAP may be involved in the formation of tumor fibrous stroma, along with activation of CAFs in HCC, giving rise to aggressive behavior. Significant correlation between EMA-expressing tumor cells and FAP-expressing CAFs and their topographic closeness suggest possible cross-talk between epithelial cells and stromal cells in the tumor microenvironment of HCC.

## References

[1] Kalluri R (2003) Basement membranes: structure, assembly and role in tumour angiogenesis. Nat Rev Cancer 3: 422–433. 12778132 10.1038/nrc1094

[2] Bhowmick NA, Chytil A, Plieth D, Gorska AE, Dumont N, et al (2004) TGF-beta signaling in fibroblasts modulates the oncogenic potential of adjacent epithelia. Science 303: 848–851. 14764882 10.1126/science.1090922

[3] Jing Y, Han Z, Zhang S, Liu Y, Wei L (2011) Epithelial-Mesenchymal Transition in tumor microenvironment. Cell Biosci 1: 29. 21880137 10.1186/2045-3701-1-29 PMC3179439

[4] Campbell NE, Kellenberger L, Greenaway J, Moorehead RA, Linnerth-Petrik NM, et al (2010) Extracellular matrix proteins and tumor angiogenesis. J Oncol 2010: 586905. 20671917 10.1155/2010/586905 PMC2910498

[5] Yang JD, Nakamura I, Roberts LR (2011) The tumor microenvironment in hepatocellular carcinoma: current status and therapeutic targets. Semin Cancer Biol 21: 35–43. 20946957 10.1016/j.semcancer.2010.10.007 PMC3050428

[6] Aboussekhra A (2011) Role of cancer-associated fibroblasts in breast cancer development and prognosis. Int J Dev Biol 55: 841–849. 22161840 10.1387/ijdb.113362aa

[7] Hafner C, Reichle A, Vogt T (2005) New indications for established drugs: combined tumor-stroma-targeted cancer therapy with PPARgamma agonists, COX-2 inhibitors, mTOR antagonists and metronomic chemotherapy. Curr Cancer Drug Targets 5: 393–419. 16178816 10.2174/1568009054863591

[8] Kalluri R, Zeisberg M (2006) Fibroblasts in cancer. Nat Rev Cancer 6: 392–401. 16572188 10.1038/nrc1877

[9] Nakasone ES, Askautrud HA, Kees T, Park JH, Plaks V, et al (2012) Imaging tumor-stroma interactions during chemotherapy reveals contributions of the microenvironment to resistance. Cancer Cell 21: 488–503. 22516258 10.1016/j.ccr.2012.02.017 PMC3332002

[10] Rasanen K, Vaheri A (2010) Activation of fibroblasts in cancer stroma. Exp Cell Res 316: 2713–2722. 20451516 10.1016/j.yexcr.2010.04.032

[11] Yang JD, Roberts LR (2010) Hepatocellular carcinoma: A global view. Nat Rev Gastroenterol Hepatol 7: 448–458. 20628345 10.1038/nrgastro.2010.100 PMC3926946

[12] Seok JY, Na DC, Woo HG, Roncalli M, Kwon SM, et al (2012) A fibrous stromal component in hepatocellular carcinoma reveals a cholangiocarcinoma-like gene expression trait and epithelial-mesenchymal transition. Hepatology 55: 1776–1786. 22234953 10.1002/hep.25570

[13] Brigstock DR (2010) Connective tissue growth factor (CCN2, CTGF) and organ fibrosis: lessons from transgenic animals. J Cell Commun Signal 4: 1–4. 19798591 10.1007/s12079-009-0071-5 PMC2821473

[14] Mazzocca A, Fransvea E, Dituri F, Lupo L, Antonaci S, et al (2010) Down-regulation of connective tissue growth factor by inhibition of transforming growth factor beta blocks the tumor-stroma cross-talk and tumor progression in hepatocellular carcinoma. Hepatology 51: 523–534. 19821534 10.1002/hep.23285

[15] von Mensdorff-Pouilly S, Snijdewint FG, Verstraeten AA, Verheijen RH, Kenemans P (2000) Human MUC1 mucin: a multifaceted glycoprotein. Int J Biol Markers 15: 343–356. 11192832 10.1177/172460080001500413

[16] Coulouarn C, Corlu A, Glaise D, Guenon I, Thorgeirsson SS, et al (2012) Hepatocyte-stellate cell cross-talk in the liver engenders a permissive inflammatory microenvironment that drives progression in hepatocellular carcinoma. Cancer Res 72: 2533–2542. 22419664 10.1158/0008-5472.CAN-11-3317 PMC3498759

[17] Ichikawa T, Yamamoto T, Uenishi T, Tanaka H, Takemura S, et al (2006) Clinicopathological implications of immunohistochemically demonstrated mucin core protein expression in hepatocellular carcinoma. J Hepatobiliary Pancreat Surg 13: 245–251. 16708303 10.1007/s00534-005-1070-4

[18] Woenckhaus M, Merk J, Stoehr R, Schaeper F, Gaumann A, et al (2008) Prognostic value of FHIT, CTNNB1, and MUC1 expression in non-small cell lung cancer. Hum Pathol 39: 126–136. 17949785 10.1016/j.humpath.2007.05.027

[19] Tamura Y, Higashi M, Kitamoto S, Yokoyama S, Osako M, et al (2012) MUC4 and MUC1 expression in adenocarcinoma of the stomach correlates with vessel invasion and lymph node metastasis: an immunohistochemical study of early gastric cancer. PLoS One 7: e49251. 23152882 10.1371/journal.pone.0049251 PMC3496698

[20] Kim SM, Oh SJ, Hur B (2012) Expression of MUC1 and MUC4 in gallbladder adenocarcinoma. Korean J Pathol 46: 429–435. 23136569 10.4132/KoreanJPathol.2012.46.5.429 PMC3490112

[21] Henriksson ML, Edin S, Dahlin AM, Oldenborg PA, Oberg A, et al (2011) Colorectal cancer cells activate adjacent fibroblasts resulting in FGF1/FGFR3 signaling and increased invasion. Am J Pathol 178: 1387–1394. 21356388 10.1016/j.ajpath.2010.12.008 PMC3070577

[22] Henry LR, Lee HO, Lee JS, Klein-Szanto A, Watts P, et al (2007) Clinical implications of fibroblast activation protein in patients with colon cancer. Clin Cancer Res 13: 1736–1741. 17363526 10.1158/1078-0432.CCR-06-1746

[23] Shi M, Yu DH, Chen Y, Zhao CY, Zhang J, et al (2012) Expression of fibroblast activation protein in human pancreatic adenocarcinoma and its clinicopathological significance. World J Gastroenterol 18: 840–846. 22371645 10.3748/wjg.v18.i8.840 PMC3286148

[24] Wikberg ML, Edin S, Lundberg IV, Van Guelpen B, Dahlin AM, et al (2013) High intratumoral expression of fibroblast activation protein (FAP) in colon cancer is associated with poorer patient prognosis. Tumour Biol 34: 1013–1020. 23328994 10.1007/s13277-012-0638-2 PMC3597266

[25] Niedermeyer J, Kriz M, Hilberg F, Garin-Chesa P, Bamberger U, et al (2000) Targeted disruption of mouse fibroblast activation protein. Mol Cell Biol 20: 1089–1094. 10629066 10.1128/mcb.20.3.1089-1094.2000 PMC85226

[26] Mathew S, Scanlan MJ, Mohan Raj BK, Murty VV, Garin-Chesa P, et al (1995) The gene for fibroblast activation protein alpha (FAP), a putative cell surface-bound serine protease expressed in cancer stroma and wound healing, maps to chromosome band 2q23. Genomics 25: 335–337. 7774951 10.1016/0888-7543(95)80157-h

[27] Yuan SF, Li KZ, Wang L, Dou KF, Yan Z, et al (2005) Expression of MUC1 and its significance in hepatocellular and cholangiocarcinoma tissue. World J Gastroenterol 11: 4661–4666. 16094706 10.3748/wjg.v11.i30.4661 PMC4615407

[28] di Mola FF, Friess H, Martignoni ME, Di Sebastiano P, Zimmermann A, et al (1999) Connective tissue growth factor is a regulator for fibrosis in human chronic pancreatitis. Ann Surg 230: 63–71. 10400038 10.1097/00000658-199907000-00010 PMC1420846

[29] Gupta S, Clarkson MR, Duggan J, Brady HR (2000) Connective tissue growth factor: potential role in glomerulosclerosis and tubulointerstitial fibrosis. Kidney Int 58: 1389–1399. 11012874 10.1046/j.1523-1755.2000.00301.x

[30] Rachfal AW, Brigstock DR (2003) Connective tissue growth factor (CTGF/CCN2) in hepatic fibrosis. Hepatol Res 26: 1–9. 12787797 10.1016/s1386-6346(03)00115-3

[31] Chen CM, Wang LF, Chou HC, Lang YD, Lai YP (2007) Up-regulation of connective tissue growth factor in hyperoxia-induced lung fibrosis. Pediatr Res 62: 128–133. 17597657 10.1203/PDR.0b013e3180987202

[32] Kwon S, Munroe X, Crawley SC, Lee HY, Spong S, et al (2007) Expression of connective tissue growth factor in pancreatic cancer cell lines. Int J Oncol 31: 693–703. 17786299

[33] Xiang ZL, Zeng ZC, Tang ZY, Fan J, He J, et al (2011) Potential prognostic biomarkers for bone metastasis from hepatocellular carcinoma. Oncologist 16: 1028–1039. 21665914 10.1634/theoncologist.2010-0358 PMC3228144

[34] Gao YB, Xiang ZL, Zhou LY, Wu ZF, Fan J, et al (2013) Enhanced production of CTGF and IL-11 from highly metastatic hepatoma cells under hypoxic conditions: an implication of hepatocellular carcinoma metastasis to bone. J Cancer Res Clin Oncol 139: 669–679. 23307318 10.1007/s00432-012-1370-4 PMC11824752

[35] Xiu M, Liu YH, Brigstock DR, He FH, Zhang RJ, et al (2012) Connective tissue growth factor is overexpressed in human hepatocellular carcinoma and promotes cell invasion and growth. World J Gastroenterol 18: 7070–7078. 23323010 10.3748/wjg.v18.i47.7070 PMC3531696

[36] Kim H, Choi GH, Na DC, Ahn EY, Kim GI, et al (2011) Human hepatocellular carcinomas with “Stemness”-related marker expression: keratin 19 expression and a poor prognosis. Hepatology 54: 1707–1717. 22045674 10.1002/hep.24559

[37] Liu S, Ginestier C, Ou SJ, Clouthier SG, Patel SH, et al (2011) Breast cancer stem cells are regulated by mesenchymal stem cells through cytokine networks. Cancer Res 71: 614–624. 21224357 10.1158/0008-5472.CAN-10-0538 PMC3100554

[38] Giannoni E, Bianchini F, Masieri L, Serni S, Torre E, et al (2010) Reciprocal activation of prostate cancer cells and cancer-associated fibroblasts stimulates epithelial-mesenchymal transition and cancer stemness. Cancer Res 70: 6945–6956. 20699369 10.1158/0008-5472.CAN-10-0785

[39] Vermeulen L, De Sousa EMF, van der Heijden M, Cameron K, de Jong JH, et al (2010) Wnt activity defines colon cancer stem cells and is regulated by the microenvironment. Nat Cell Biol 12: 468–476. 20418870 10.1038/ncb2048

[40] Vermeulen L, de Sousa e Melo F, Richel DJ, Medema JP (2012) The developing cancer stem-cell model: clinical challenges and opportunities. Lancet Oncol 13: e83–89. 22300863 10.1016/S1470-2045(11)70257-1

